# MDM2 gene amplification and expression in non-small-cell lung cancer: immunohistochemical expression of its protein is a favourable prognostic marker in patients without p53 protein accumulation.

**DOI:** 10.1038/bjc.1997.221

**Published:** 1997

**Authors:** M. Higashiyama, O. Doi, K. Kodama, H. Yokouchi, T. Kasugai, S. Ishiguro, K. Takami, T. Nakayama, I. Nishisho

**Affiliations:** Department of Thoracic Surgery, Osaka Medical Center for Cancer and Cardiovascular Diseases (formerly, Center for Adult Diseases, Osaka), Higashinariku, Japan.

## Abstract

**Images:**


British Journal of Cancer (1997) 75(9), 1302–1308
© 1997 Cancer Research Campaign

### *MDM2* gene amplification and expression in non-small-cell lung cancer: immunohistochemical expression of its protein is a favourable prognostic marker in patients without p53 protein accumulation

**M Higashiyama[1], O Doi[1], K Kodama[1], H Yokouchi[1], T Kasugai[2], S Ishiguro[2], K Takami[3], T Nakayama[3] and I Nishisho[3]**

Departments of [1]Thoracic Surgery and [2]Pathology, Osaka Medical Center for Cancer and Cardiovascular Diseases (formerly, Center for Adult Diseases, Osaka); [3]Division of Clinical Genetics, Department of Medical Genetics, Biomedical Research Center, Osaka University Medical School, Osaka, Japan

**Summary** MDM2 is an oncoprotein that inhibits p53 tumour-suppressor protein. Amplification of the *MDM2* gene and overexpression of its protein have been observed in some human malignancies, and these abnormalities have a role in tumorigenesis through inactivation of p53 function. To determine the clinicopathological and prognostic value of MDM2 abnormalities in non-small-cell lung cancer (NSCLC), *MDM2* gene amplification and its protein expression status were analysed in surgically resected materials. *MDM2* gene amplification was detected in only 2 (7%) of the 30 tested patients. MDM2 protein was found immunohistochemically in a total of 48 (24%) of the 201 patients. MDM2 protein was slightly frequently observed in patients with adenocarcinoma, but its presence or absence was not associated with clinicopathological factors such as T-factor, N-factor, stage, tumour size, differentiation or p53 protein status. Overall, MDM2-positive patients tended to have a better prognosis ($P = 0.062$). In particular, among immunohistochemically p53-negative patients ($n = 110$), those with positive MDM2 protein expression showed significantly better prognosis ($P = 0.039$) and, in a multivariate analysis, MDM2 protein status was a favourable prognostic factor ($P = 0.037$). In contrast, among p53-positive patients ($n = 91$), there was no difference in prognosis depending on MDM2 protein status. Thus, in the NSCLC patients studied, *MDM2* gene amplification was a minor event, but expression of its protein, which was often observed immunohistochemically, was a favourable prognostic marker, especially among patients without p53 protein accumulation. Further study is needed to determine how MDM2 protein expression results in a better prognosis.

**Keywords:** *MDM2* gene; non-small-cell lung cancer; p53; prognosis; immunohistochemistry; amplification; fluorescence-based polymerase chain reaction single-strand conformation polymorphism

The *MDM2* gene was originally identified and cloned by amplification in a transformed tumorigenic Balb/c 3T3 fibroblast cell line (Cahilly-Snyder et al, 1987; Fakharzadeh et al, 1991; Oliner et al, 1992). Its product, p90, is now considered to form a tight complex with both the wild-type and mutant p53 tumour-suppressor gene protein and to inactivate wild-type p53 function by masking the N-terminal acidic transactivating domain of p53 protein, indicating that abnormalities of the *MDM2* gene may be closely associated with tumorigenesis and/or tumour development (Olson et al, 1993; Haines et al, 1994). Indeed, *MDM2* gene amplification and overexpression of its product have been described in several types of malignancies in humans. However, the clinicopathological role of these abnormalities has yet to be determined.

In lung cancer, p53 abnormalities have been well examined but, to our knowledge, MDM2 abnormalities in this disease have been reported only by Marchetti et al (1995*a*). However, because of the relatively small number of samples tested in their study, its clinicopathological and prognostic significance is as yet unknown. Hence, because p53 abnormalities in this disease may be essential for biological and clinical characteristics of the tumour, we examined MDM2 abnormality status, especially in those patients without p53 abnormalities. *MDM2* gene amplification and its protein expression were examined in a series of surgically resected non-small-cell lung cancer (NSCLC) cases in association with clinicopathological parameters, p53 protein accumulation and prognosis.

*Received 8 July 1996*
*Revised 7 October 1996*
*Accepted 23 October 1996*

*Correspondence to:* M Higashiyama, Department of Thoracic Surgery, Osaka Medical Center for Cancer and Cardiovascular Diseases, Nakamichi 1–3–3, Higashinariku, Osaka, 537, Japan

### MATERIALS AND METHODS

### Clinical materials

For immunohistochemical analysis of MDM2 protein expression and p53 accumulation, formalin-fixed paraffin-embedded tissue blocks of primary tumours removed surgically between July 1989 and June 1995 at the Department of Thoracic Surgery, Osaka Medical Center for Cancer and Cardiovascular Diseases (formerly the Center for Adult Diseases, Osaka), were obtained from 201 NSCLC patients. For amplification analysis of the *MDM2* gene, 30 fresh tissue samples were immediately frozen at the time of the operation and stored at –80°C until DNA extraction.

Of the 201 patients, 146 were men and 48 were women; they were aged between 35 and 83 years (mean 63.4 years). The histological type was adenocarcinoma in 116 patients, squamous cell carcinoma in 71, large-cell carcinoma in 12 and adenosquamous cell carcinoma in two. The pathological staging was according to

**Figure 1** Immunostaining of strongly positive MDM2 protein expression in NSCLC (++). Almost all nuclei of cancer cells (percentage of positive cells: **A**, 75%; **B**, 99%) show positive immunoreactivity for MDM2 protein. (**A**) Squamous cell carcinoma (original magnification × 33). (**B**) Adenocarcinoma (original magnification ×40)

**Figure 2** Amplification of *MDM2* gene in NSCLCs. (**A**) Southern blot hybridization with the MDM2 probe. (**B**) Southern blot hybridization with the pYNH132 probe for internal control. *MDM2* gene amplification was observed in case no. 24 (lane 1) and case no. 3 (lane 4)

the international TNM staging system (Mountain, 1986): stage I in 115, stage II in 23, stage IIIA in 57 and stage IIIB in six. All patients underwent potentially curative operations. The median post-operative follow-up of the patients was 839 days (range 32–2469 days). Post-operative survival curves were constructed using the Kaplan–Meier method.

### *MDM2* amplification and *p53* mutation analysis

Southern blot analysis was performed to detect amplification of the *MDM2* gene, as described previously (Takami et al, 1994). The human MDM2 cDNA clone (c14-2; nt 1–949), which was kindly provided by Drs B Vogelstein and KW Kinzler, was used as a hybridization probe. pYNH132 on chromosome 6p, pLYNZ9.1 on chromosome 2p and pMCA1-1 on chromosome 15q were used as internal diploid standards (kindly provided by Dr Y Nakamura). Briefly, 5 μg of high molecular weight DNA derived from tumour tissues was digested with *Eco*R I and then electrophoresed on 0.8% agarose gel followed by transfer to a nylon filter. The DNAs on the filters were sequentially hybridized with the *MDM2* gene probe, c14-2, and three control probes, pYNH132, pLYNZ9.1 and pMCA1-1. The intensity of the hybridization signals was measured by densitometry. The relative signal intensities of the *MDM2* gene were calculated by comparing the ratio of MDM2 to three control probes and, when the samples showed a more than two fold increase in signal intensity of *MDM2*, they were hybridized with two probes, pYNH15 on chromosome 12q and pHu*a*2M9 on chromosome 12p, to determine whether or not the increase was due to non-specific polysomies of the chromosome.

Mutations of the *p53* gene were examined using fluorescence-based polymerase chain reaction single-strand conformation polymorphism (PCR-FSSCP), as described previously by Katsuragi et al (1995). This technique was used for the detection of point mutations in the *p53* gene in exons 5, 6, 7 and 8 by an automated DNA sequencer and software.

### Immunohistochemical analysis

Sections were cut at 4 μm, dewaxed and rehydrated through a graded ethanol series. Before staining, sections were pretreated with microwave irradiation for antigen retrieval, as described previously (Cattoretti et al, 1992; Marchetti et al, 1995*b*; McCann et al, 1995; Ofner et al, 1995). Incubation with the primary antibodies (monoclonal MDM2 antibody, IF-2, Oncogene Science, USA, and monoclonal p53 antibody, DO-7, Novocastra Laboratories, UK),

© Cancer Research Campaign 1997

Table 1 MDM2 and p53 abnormalities in NSCLC

| No. | Histology stage | MDM2 | | p53 | |
|---|---|---|---|---|---|
| | | Amplification | Protein | Mutation | Protein |
| 1 | Ad IIIB | – | – | – | + |
| 2 | Ad IIIB | – | – | NT | ++ |
| 3 | Ad IIIA | +(5.5-fold) | ++ | – | ++ |
| 4 | Ad IIIA | – | + | NT | – |
| 5 | Ad IIIA | – | ++ | +(Exon6) | ++ |
| 6 | Ad IIIA | – | – | NT | – |
| 7 | Ad IIIA | – | – | NT | – |
| 8 | Ad IIIA | – | – | NT | – |
| 9 | Ad II | – | + | – | + |
| 10 | Ad II | – | – | NT | ++ |
| 11 | Ad II | – | – | NT | ++ |
| 12 | Ad I | – | + | – | – |
| 13 | Ad I | – | – | – | ++ |
| 14 | Ad I | – | + | – | – |
| 15 | Ad I | – | + | – | – |
| 16 | Ad I | – | – | – | – |
| 17 | Ad I | – | – | – | + |
| 18 | Ad I | – | – | NT | – |
| 19 | Sq IIIB | – | – | – | ++ |
| 20 | Sq IIIA | – | + | +(Exon8) | + |
| 21 | Sq IIIA | – | – | NT | ++ |
| 22 | Sq IIIA | – | – | NT | – |
| 23 | Sq II | – | + | +(Exon5) | ++ |
| 24 | Sq I | +(2.7-fold) | + | – | ++ |
| 25 | Sq I | – | – | +(Exon7) | + |
| 26 | Sq I | – | – | NT | + |
| 27 | Sq I | – | + | NT | – |
| 28 | Sq I | – | – | NT | + |
| 29 | La IIIA | – | + | NT | ++ |
| 30 | La I | – | – | NT | – |
| Positive patients/tested patients | | 2/30 (7%) | 12/30 (40%) | 4/15 (27%) | 18/30 (60%) |

Ad, adenocarcinoma; Sq, squamous cell carcinoma; La, large-cell carcinoma; NT, not tested.

Table 2 Relationship between MDM2 oncoprotein and clinicopathological parameters in 201 NSCLC patients undergoing potentially curative operation

| | MDM2 protein status | | | P-value |
|---|---|---|---|---|
| | – (n = 153) 76% | + (n =38) 19% | ++ (n =10) 5% | |
| Gender | | | | |
| Male | 117 | 24 | 5 | |
| Female | 36 | 14 | 5 | 0.07 |
| Age (mean year) | 63.2 | 65.0 | 61.2 | NS |
| T-factor | | | | |
| T1 | 42 | 13 | 5 | |
| T2 | 80 | 19 | 4 | |
| T3,4 | 31 | 6 | 1 | NS |
| N-factor | | | | |
| N0 | 103 | 27 | 7 | |
| N1 | 24 | 4 | 1 | |
| N2,3 | 26 | 7 | 2 | NS |
| Stage | | | | |
| I | 85 | 23 | 7 | |
| II | 19 | 3 | 1 | |
| IIIA | 44 | 11 | 2 | |
| IIIB | 5 | 1 | 0 | NS |
| Tumour size (mm) | | | | |
| ≤ 20 | 22 | 6 | 2 | |
| > 20 to ≤ 40 | 82 | 17 | 7 | |
| > 40 to ≤ 60 | 32 | 9 | 0 | |
| > 60 | 17 | 6 | 1 | NS |
| Histology | | | | |
| Ad | 84 | 23 | 9 | |
| Sq | 58 | 12 | 1 | |
| La | 10 | 2 | 0 | |
| As | 1 | 1 | 0 | 0.09 (Ad vs non-Ad[a]) |
| Differentiation | | | | |
| Well | 40 | 13 | 3 | |
| Moderate | 73 | 18 | 6 | |
| Poor | 40 | 7 | 1 | NS |
| p53 protein status | | | | |
| p53 – | 84 | 21 | 5 | |
| p53 + | 31 | 11 | 2 | |
| p53 ++ | 38 | 6 | 3 | NS |

[a]Ad, adenocarcinoma; non-Ad, non-adenocarcinoma, including Sq (squamous cell carcinoma), La (large-cell carcinoma) and As (adenosquamous cell carcinoma).

the enzyme colour reaction, haematoxylin counterstaining and mounting were carried out as described elsewhere (Foulkes et al, 1995; Marchetti et al, 1995*a* and *b*; McCann et al, 1995; Ofner et al, 1995; Matsumura et al, 1996).

Immunostaining results were assessed, taking into account the cancer cells whose nuclei showed positive immunoreactivity for MDM2 (Figure 1) or p53 protein. The percentage of immunoreactive nuclei was evaluated by scanning the whole section at medium and high magnification and by counting at least 500 cells in the most densely stained tumour areas. The patients were classified into three groups: a strongly positive group (++), with more than 50% positive cancer cells in the tissue; a weakly positive group (+), with 10–50% positive cancer cells; and a negative group (–) with less than 10% or no positive cancer cells (McCann et al, 1995).

### Statistical analysis

The chi-square test was applied for statistical analysis. For survival data, statistical significance was analysed using the log-rank test. Variables related to survival were analysed using Cox's proportional hazards regression model with SAS software (Statistical Analysis Institute, Cary, NC, USA). $P < 0.05$ was considered to be significant and $0.05 \leq P < 0.10$ was considered to be marginally significant.

### RESULTS

### *MDM2* amplification and *p53* mutation

Of the 30 patients tested, only two (7%) showed *MDM2* gene amplification (Figure 2 and Table 1): one had stage IIIA adenocarcinoma and another stage I squamous cell carcinoma. Amplification grade was 5.5-fold in the former and 2.7-fold in the latter. Both showed MDM2 protein expression by immunohistochemical analysis (strongly positive in the former and weakly positive in the latter, percentage of positive cells being 90% and 48% respectively). In contrast, ten patients showed MDM2 protein expression with no evidence of its gene amplification.

In the present series, 4 (27%) of the 15 patients showed p53 mutations (exon 5, 6, 7 or 8 in each, Table 1) using the PCR-FSSCP

© Cancer Research Campaign 1997

**Figure 3** Post-operative survival curves according to MDM2 protein expression in 201 NSCLC patients. Overall, among the three groups (– vs + vs ++), there is no statistical difference in post-operative survival curves ($P = 0.163$), but MDM2-positive patients, including those with strong (++) and weak (+) expression, show marginally better prognosis than MDM2-negative patients ($P = 0.062$)

**Table 3** *P*-value from univariate analysis using the log-rank test for 201 NSCLC patients undergoing potentially curative operation

| Variables | *P*-value |
|---|---|
| Gender | |
| Male vs Female | 0.260 |
| Age (year) | |
| ≤60 vs 61≤ | 0.191 |
| T-factor | |
| T1 vs T2 vs T3,4 | 0.048 |
| N-factor | |
| N0 vs N1 vs N2,3 | < 0.0001 |
| Stage | |
| I vs II vs III | < 0.0001 |
| Tumour size (mm) | |
| ≤30 vs 31 ≤ | 0.079 |
| Histology | |
| Ad vs non-Ad* | 0.752 |
| Differentiation | |
| Well vs moderate vs poor | 0.002 |
| p53 protein status | |
| – vs + vs ++ | 0.449 |
| – vs +, ++ | 0.746 |
| MDM2 protein status | |
| – vs + vs ++ | 0.163 |
| – vs +, ++ | 0.062 |

[a]Ad, adenocarcinoma; non-Ad, non-adenocarcinoma, including Sq (squamous cell carcinoma), La (large-cell carcinoma) and As (adenosquamous cell carcinoma).

method. Although the two patients with *MDM2* gene amplification and MDM2 protein expression also showed p53 protein accumulation, no p53 mutation was observed.

### Association between MDM2 protein expression and clinicopathological parameters

Of the 201 patients tested, a total of 48 (24%) showed positive immunostaining for MDM2 protein within the tumour tissue: ten (5%) were in the strongly positive group and 38 (19%) were in the weakly positive group. The median percentage of MDM2-positive cells was 75% (mean ± s.d. 78 ± 17%, range 55–99%) in the former and 30% (mean ± s.d. 28 ± 7%, range 11–48%) in the latter.

**Table 4** Multivariate analysis of Cox's proportional hazards model in 110 p53-negative patients

| Variables | Coefficient | s.e. | $\chi^2$ | *P*-value |
|---|---|---|---|---|
| T-factor | | | | |
| T1,2 vs T3,4 | 0.228 | 0.219 | 1.085 | 0.299 |
| N-factor | | | | |
| No vs N1,2,3 | 0.955 | 0.204 | 21.990 | <0.0001 |
| Differentiation | | | | |
| Well, moderate vs poor | 1.717 | 0.264 | 0.423 | 0.517 |
| MDM2 protein | | | | |
| – vs +, ++ | –0.568 | 0.269 | 4.477 | 0.037 |

In comparison with the non-adenocarcinoma type, including squamous cell carcinoma, large-cell carcinoma and adenosquamous cell carcinoma, adenocarcinoma type marginally frequently showed MDM2 protein expression ($P = 0.09$). However, MDM2 protein status was not associated with the representative clinicopathological parameters, such as T-classification (T-factor), nodal involvement (N-factor), stage, tumour size or differentiation (Table 2).

p53 protein accumulation was seen in a total of 91 patients (45%): 47 (23%) patients showed strongly positive immunoreactivity for p53 protein (median percentage of positive cells 89%, mean ± s.d. 88 ± 5%, range 55–100%) and 44 (22%) showed weak positivity (median percentage of positive cells 28%, mean ± s.d. 33 ± 8%, range 13–49%). There was no association between MDM2 protein expression and p53 protein accumulation status in the present series (Table 2). The immunohistochemical distribution of positive cells in the tumour tissue of both MDM2- and p53-positive patients was diverse: some patients showed an almost similar pattern of distribution, while others showed mosaic pattern of MDM2 protein expression and p53 protein accumulation in the tissue.

### Post-operative prognosis

Decending on MDM2 protein expression status, post-operative overall survival was analysed for 201 patients (Figure 3). Overall, there was no statistically significant difference in prognosis among the three groups ($P = 0.163$), but MDM2-positive patients, including those strongly and weakly positive, showed slightly better prognosis than MDM2-negative patients ($P = 0.062$). In addition to MDM-2 protein status, T-factor, N-factor, stage, tumour size and differentiation were also significantly or marginally significantly associated with prognosis (Table 3). p53 protein status showed no influence on prognosis in the present series.

Considering a biophysiological function of MDM2 and p53 proteins (Olson et al, 1993; Haines et al, 1994), survival was separately analysed according to p53 protein status. In the p53-negative group (110 patients), MDM2-positive patients, including those strongly and weakly positive, showed a significantly more favourable prognosis than MDM2-negative patients (Figure 4A, $P = 0.039$). In particular, even among the p53-negative group with stage I disease (64 patients), a similar result was obtained (Figure 4B, $P = 0.049$). In a multivariate analysis of the p53-negative group (Table 4), the *P*-value of MDM2 protein expression was significant for survival ($P = 0.037$), in addition to N-factor. However, among the p53-positive group (91 patients), there was no difference in the post-operative survival curve with the MDM2 protein status (Figure 4C, $P = 0.556$).

**Figure 4** Post-operative survival curves according to MDM2 protein expression among p53-negative group (**A**), p53-negative group with stage I disease (**B**) and among p53-positive group (**C**). In the p53-negative group (110 patients), the MDM2-positive patients (++ and +) showed significantly more favourable prognosis than the MDM2-negative patients (**A**, $P = 0.039$), even in the p53-negative group with stage I disease (64 patients) (**B**, $P = 0.049$). In contrast, in the p53-positive group (91 patients), there was no difference in post-operative survival curves (**C**, $P = 0.556$)

### DISCUSSION

*MDM2* gene amplification has been described in several types of human sarcomas (Leach et al, 1993; Florenes et al, 1994; Bueso-Ramos et al, 1995; Nakayama et al, 1995), including 15–36% of soft tissue sarcomas and 10–15% of osteosarcomas. Similarly, this amplification has been detected in 8–10% of brain tumours (Reifenberger et al, 1993). *MDM2* gene amplification has also been reported in human cancers, such as breast cancer, in which its incidence is 4–13% of the primary tumours (Marchetti et al, 1995*b*; McCann et al, 1995), and oesophageal cancer, in which the incidence is as high as 18% (Shibagaki et al, 1995); but overall in other cancers, including urinary bladder tumours (Lianes et al, 1994), cervical cancers (Kessis et al, 1993; Ikenberg et al, 1995), head and neck tumours (Waber et al, 1993), some paediatric tumours (Waber et al, 1993) and urothelial cancers (Habuchi et al, 1994), this event is now considered to be rather infrequent. In the NSCLC cases studied here, its incidence was only 7% (2 of 30 patients tested), compatible with that (6%) reported by Marchetti et al (1995*a*), and, in addition, its amplification grade was relatively low (5.5-fold and 2.7-fold) in comparison with the other tumours, indicating that amplification of this oncogene in this disease may also be a relatively infrequent event through tumorigenesis and tumour development. Interestingly, Marchetti et al (1995*a*) emphasized that *MDM2* gene amplification was observed only in patients with adenocarcinoma but, in the present study, one patient with squamous cell carcinoma showed *MDM2* gene amplification.

In immunohistochemical studies using monoclonal anti-MDM2 protein antibody, the incidence of MDM2 immunohistochemical (over)expression in human cancers has been reported to be 30% in bladder cancers (Lianes et al, 1994), 20% in colorectal cancers (Ofner et al, 1995), 3% in ovarian cancers (Foulkes et al, 1995), 40% in oral carcinoma (Matsumura et al, 1996) and 22–41% in breast cancers (Marchetti et al, 1995*b*; McCann et al, 1995). In NSCLC, whereas Marchetti et al (1995*a*) reported that *MDM2* oncogene product expression was detected immunohistochemically only in three (6%) patients with gene amplification, it was detected in the present study in 24% of patients, independently of *MDM2* gene amplification status. This difference in incidence reported between Marchetti et al (1995*a*) and ourselves is probably caused by the use of frozen sections in the former study, whereas paraffin sections, possibly larger in size, were used in the present study. In addition, the criteria for the positivity of immunostaining may have been another related factor. Thus, when considering together our findings on NSCLC, we believe that its expression (in contrast to its gene amplification) is not a rare, but a more common, event in human cancer tissues.

Although Marchetti et al (1995*a*) reported that MDM2 protein was not detected in patients with squamous cell carcinoma, 13 patients in this study, including 12 with weak and one with strong expression, expressed its protein. Recently, Matsumura et al (1996) reported that MDM2 protein was observed immunohistochemically in 40% of oral squamous cell carcinomas. Therefore, considering these results together, *MDM2* gene product expression does occur in adenocarcinoma type in NSCLC, slightly frequently.

The relationship between *MDM2* gene amplification and increased expression of the product appears to be complicated and is not completely understood. In fact, in several unique cases, the gene was amplified in the absence of increased expression (McCann et al, 1995). Although we did not examine the *MDM2* gene mRNA levels in NSCLC, one case exhibiting gene amplification (case no. 3) showed strong expression of its product (percentage of positive cells 90%) and another (case no. 24) showed weak expression (percentage of positive cells 48%). Marchetti et al (1995*a*) also reported that all of the patients in their study with *MDM2* gene amplification did not show strong expression of its protein.

In the present study, there was no significant association between MDM2 expression and tumour-staged parameters (Table 2) and, even in regard to *MDM2* gene amplification, one case was in stage I and another in stage IIIA. There was also no such association in the three patients with positive amplification and overexpression described by Marchetti et al (1995*a*). In addition, MDM2 protein status did not appear to be associated with p53 protein accumulation status (Table 2), and there was no definite distribution of positive cells in the tumour tissue of both MDM2- and p53-positive patients. Thus, the combination of *MDM2* and *p53*

© Cancer Research Campaign 1997

abnormalities in NSCLC may be not so simple. However, only in the two patients with *MDM2* amplification, no *p53* gene mutation was detected in spite of strongly positive p53 protein accumulation; this observation is compatible with that of Marchetti et al (1995*a*). Considering that the antibody recognizes both the wild-type and the mutant forms of p53 protein, it is possible that immunohistochemically detected protein in such patients is wild-type p53, which may be stabilized and accumulated by MDM2 protein expression (Keleti et al, 1996).

The prognostic value of MDM2 protein expression is observed only among p53-negative patients, but not among p53-positive patients. The observation that MDM2-positive patients showed marginally better prognosis than MDM2-negative patients, on the whole, reflects the findings among p53-negative patients. Thus, it is concluded that MDM2 protein status is a useful prognostic marker only in such patients. Considering a biophysiological function of MDM2 and p53 proteins (Olson et al, 1993; Haines et al, 1994), mutant-type p53 itself may have lost its p53 function, leading to the speculation that MDM2 abnormalities have little or no effect on the p53-mediated pathway. The findings observed among p53-positive patients in this study support this hypothesis. In contrast, we had hypothesized that MDM2 abnormalities are an alternative mechanism, escaping from p53-regulated growth control in wild-type p53 tumours in the same fashion as in mutant-p53 tumours, but the present findings obtained in NSCLCs appear to be rather paradoxical.

The reason for the present clinical outcome among the p53-negative patients is unknown. In this respect, it was recently reported that the *MDM2* gene encodes a number of alternatively spliced mRNAs that give rise to proteins ranging in size from 40 kDa to 90 kDa. Several investigators have described not only a p90 protein, the original form, but also the representative forms, p57-58, p74, p76 and p85 proteins as the *MDM2* gene products in various types of tumours (Olson et al, 1993; Haines et al, 1994; Landers et al, 1994; Bueso-Ramos et al, 1995; Gudas et al, 1995). In particular, it is noteworthy that the variant forms p74 and p76, lacking the N-terminal protein domain of p90 protein, do not inhibit p53 protein (Olson et al, 1993; Haines et al, 1994). The antibody IF2, used in the present study, enables detection of such forms as p90, p74, p76 and p57-58 (Haines et al, 1994; Gudas et al, 1995). Therefore, some immunohistochemically detected MDM2-positive patients may have been included as showing a different function of variant MDM2 protein, e.g. p74 or p75, from the original MDM2 protein, p90. Secondly, p53 abnormalities examined by immunohistochemistry are not always consistent with those examined by analysis of its gene. In fact, several patients even with strongly positive p53 accumulation showed no p53 mutation by the PCR-FSSCP method (Table 1). Conversely, it is possible that some p53-negative patients may have been included as those with some p53 abnormalities at the gene level. The present prognostic analysis was firmly based on immunohistochemistry for tumour phenotype and, therefore, further study is needed to elucidate the mechanism underlying the apparently contradictory effects of MDM2 protein on prognosis.

In other tumours, the prognostic value of MDM2 abnormalities remains controversial. In breast cancers, MDM2 overexpression is strongly associated with oestrogen receptor expression, suggesting that MDM2 expression status may also be a favourable prognostic factor (Sheikh et al, 1993; Takami et al, 1994; Marchetti et al, 1995*b*; McCann et al, 1995; Gudas et al, 1995). In bladder cancer, Lianes et al (1994) reported that MDM2 overexpression was observed in patients with relatively early-staged and low-grade tumours, suggesting that MDM2 overexpression may be an early event or possibly a favourable factor associated with low-grade malignancy, although there have been no reports clearly describing its association with prognosis. On the other hand, in oesophageal cancer, *MDM2* gene amplification has been described as being a rather unfavourable prognostic factor (Schibagaki et al, 1995). *MDM2* overexpression in leukaemias also appears to be associated with unfavourable chromosomal abnormalities (Bueso-Ramos et al, 1993). Thus, the influence of *MDM2* gene abnormalities on tumour malignancy may appear to be different when studied with tumour tissues. Further study is needed considering p53 abnormalities in these tumours to determine the prognostic value of *MDM2* abnormalities.

### ACKNOWLEDGEMENTS

We thank Drs KW Kinzler and B Vogelstein for their generous gift of the MDM2 cDNA probe and Dr Y Nakamura for contributing pYNH132, pLYNZ9.1 and pMCA1-1. We also thank Mrs Yoko Funai and Yumiko Koyanagi for technical assistance throughout the present study.

### REFERENCES

Bueso-Ramos C, Yang Y, deLeon E, McCown P, Stass SA and Albitar M (1993) The human MDM-2 oncogene is overexpressed in leukemias. *Blood* **82**: 2617–2623

Bueso-Ramos C, Yang Y, Manshouri T, Feltz L, Ayala A, Glassman AB and Albitar M (1995) Molecular abnormalities of MDM-2 in human sarcomas. *Int J Oncol* **7**: 1043–1048

Cahilly-Snyder L, Yang-Feng T, Francke U and George DL (1987) Molecular analysis and chromosomal mapping of amplified genes isolated from a transformed mouse 3T3 cell line. *Somatic Cell Mol Genet* **13**: 235–244

Cattoretti G, Becker MHG, Key G, Duchrow M, Schluter C, Galle J and Gerdest J (1992) Monoclonal antibodies against recombinant parts of the Ki-67 antigen (MIB1 and MIB3) detect proliferating cells in microwave-processed formalin-fixed paraffin sections. *J Pathol* **168**: 357–363

Fakharzadeh SS, Trusko SP and George DL (1991) Tumorigenic potential associated with enhanced expression of a gene that is amplified in a mouse tumor cell line. *EMBO J* **10**: 1565–1569

Florenes VA, Moelandsmo GM, Forus A, Andreassen A, Myklebost O and Fodstad O (1994) MDM2 gene amplification and transcript levels in human sarcomas: relationship to TP53 gene status. *J Natl Cancer Inst* **86**: 1297–1302

Foulkes WD, Stamp GWH, Afzal N, McFarlane CP, Trowsdale J and Campbell (1995) MDM2 overexpression is rare in ovarian carcinoma irrespective of TP53 mutation status. *Br J Cancer* **72**: 883–888

Gudas JM, Hguyen H, Klein RC, Katayose D, Seth P and Cowan KH (1995) Differential expression of multiple MDM2 messenger RNAs and proteins in normal and tumorigenic breast epithelial cells. *Clin Cancer Res* **1**: 71–80

Haines DS, Landers JE, Engle LJ and George DL (1994) Physical and functional interaction between wild-type p53 and mdm2 proteins. *Mol Cell Biol* **14**: 1171–1178

Habuchi T, Kinoshita H, Yamada H, Kakehi Y, Ogawa O, Wu W-J, Takahashi R, Sugiyama T and Yoshida O (1994) Oncogene amplification in urothelial cancers with p53 gene mutation or MDM2 amplification. *J Natl Cancer Inst* **86**: 1331–1335

Ikenberg H, Matthay K, Schmitt B, Bauknecht T, Kiechle-Schwarz M, Goppinger A and Pfleiderer A (1995) p53 mutation and MDM2 amplification are rare even in human papillomavirus-negative cervical carcinomas. *Cancer* **76**: 57–66

Katsuragi K, Chiba W, Matsubara Y, Ikeda S, Ueta C and Kinoshita M (1995) A sensitive and high-resolution method for the detection of mutations in the p53 gene using fluorescence-based PCR-SSCP. *Biomedical Res* **16**: 273–279

Keleti J, Quezado MM, Abaza MM, Raffeld M and Tsokos M (1996) The MDM2 oncoprotein is overexpressed in rhabdomyosarcoma cell lines and stabilizes wild-type p53 protein. *Am J Pathol* **149**: 143–151

© Cancer Research Campaign 1997

Kessis TD, Slebos RJ, Han SM, Shah K, Bosch XF, Munoz N, Hedrick L and Cho KR (1993) p53 gene mutations and MDM2 amplification are uncommon in primary carcinomas of the uterine cervix. *Am J Pathol* **143**: 1398–1405

Landers J, Haines DS, Stauss JF III and George DL (1994) Enhanced translation: a novel mechanism of mdm2 oncogene overexpression identified in human tumor cells. *Oncogene* **9**: 2745–2750

Leach FS, Tokino T, Meltzer P, Burrell M, Oliner JD, Smith S, Hill DE, Sidransky D, Kinzler KW and Vogelstein B (1993) p53 mutation and MDM2 amplification in human soft tissue sarcomas. *Cancer Res* **53**: 2231–2234

Lianes P, Orlow I, Zhang Z-F, Oliva MR, Sarkis AS, Reuter VE and Cordon-Cardo C (1994) Altered patterns of MDM2 and TP53 expression in human bladder cancer. *J Natl Cancer Inst* **86**: 1325–1330

Marchetti A, Buttitta F, Pellegrini S, Merlo G, Chella A, Angeletti A and Bevilacqua G (1995*a*) mdm2 gene amplification and overexpression in non-small cell lung carcinomas with accumulation of the p53 protein in the absence of p53 gene mutations. *Diagn Mol Pathol* **4**: 93–97

Marchetti A, Buttitta F, Girlando S, Palma PD, Pellegrini SW, Fina P, Doglioni C, Bevilacqua G and Barbareschi M (1995*b*) mdm2 gene alterations and mdm2 protein expression in breast carcinomas. *J Pathol* **175**: 31–38

Matsumura T, Yoshihara Y, Kimura T, Shintani S and Alcalde RE (1996) p53 and MDM2 expression in oral squamous cell carcinoma. *Oncology* **53**: 308–312

McCann AH, Kirley A, Carney DN, Corbally N, Magee HM, Keating G and Dervan PA (1995) Amplification of the MDM2 gene in human breast cancer and its association with MDM2 and p53 protein status. *Br J Cancer* **71**: 981–985

Mountain CF (1986) A new international staging system for lung cancer. *Chest* **89**: 225–232

Nakayama T, Toguchida J, Wadayama B, Kanoe H, Kotoura Y and Sasaki M (1995) MDM2 gene amplification in bone and soft-tissue tumors: association with tumor progression in differentiated adipose-tissue tumors. *Int J Cancer* **64**: 342–346

Ofner D, Maier H, Riedmann B, Holzberger P, Nogler M, Totsch M, Bankfalvi A, Winde G, Bocker W and Schmid KW (1995) Immunohistochemically detectable p53 and mdm-2 oncoprotein expression in colorectal carcinoma: prognostic significance. *J Clin Pathol*: *Mol Pathol* **48**: M12–M16

Oliner JD, Kinzler KW, Meltzer PS, George DL and Vogelstein B (1992) Amplification of a gene encoding a p53-associated protein in human sarcomas. *Nature* **358**: 80–83

Olson DC, Marechal V, Momand J, Chen J, Romocki C and Levine AJ (1993) Identification and characterization of multiple mdm-2 proteins and mdm-2-p53 protein complexes. *Oncogene* **8**: 2353–2360

Qian W, Hu L-F, Chen F, Wang Y, Magnusson KP, Kashuba E, Klein G and Wiman KG (1995) Infrequent MDM2 gene amplification and absence of gross WAF-1 gene alterations in nasopharyngeal carcinoma. *Eur J Cancer* **31B**: 328–332

Reifenberger G, Liu L, Ichimura K, Schmidt EE and Collins VP (1993) Amplification and overexpression of the MDM2 gene in a subset of human malignant gliomas without p53 mutations. *Cancer Res* **53**: 2736–2739

Sheikh MS, Shao Z-M, Hussain A and Fontana JA (1993) The p53-binding protein MDM2 gene is differentially expressed in human breast carcinoma. *Cancer Res* **53**: 3226–3228

Shibagaki I, Tanaka H, Shimada Y, Wagata T, Ikenaga M, Imamura M and Ishizaki K (1995) p53 mutation, murine double minute 2 amplification, and human papillomavirus infection are frequently involved but not associated with each other in esophageal squamous cell carcinoma. *Clin Cancer Res* **1**: 769–773

Takami K, Inui H, Nagayama K, Watatani M, Yasutomi M, Kurahashi H, Mori T, Takai S and Nishisho I (1994) Low grade amplification of MDM2 gene in a subset of human breast cancers without p53 alterations. *Breast Cancer* **1**: 95–102

Waber PG, Chen J and Nisen PD (1993) Infrequency of MDM2 gene amplification in pediatric solid tumors and lack of association with p53 mutations in adult squamous cell carcinomas. *Cancer Res* **53**: 6028–6030

*© Cancer Research Campaign 1997*


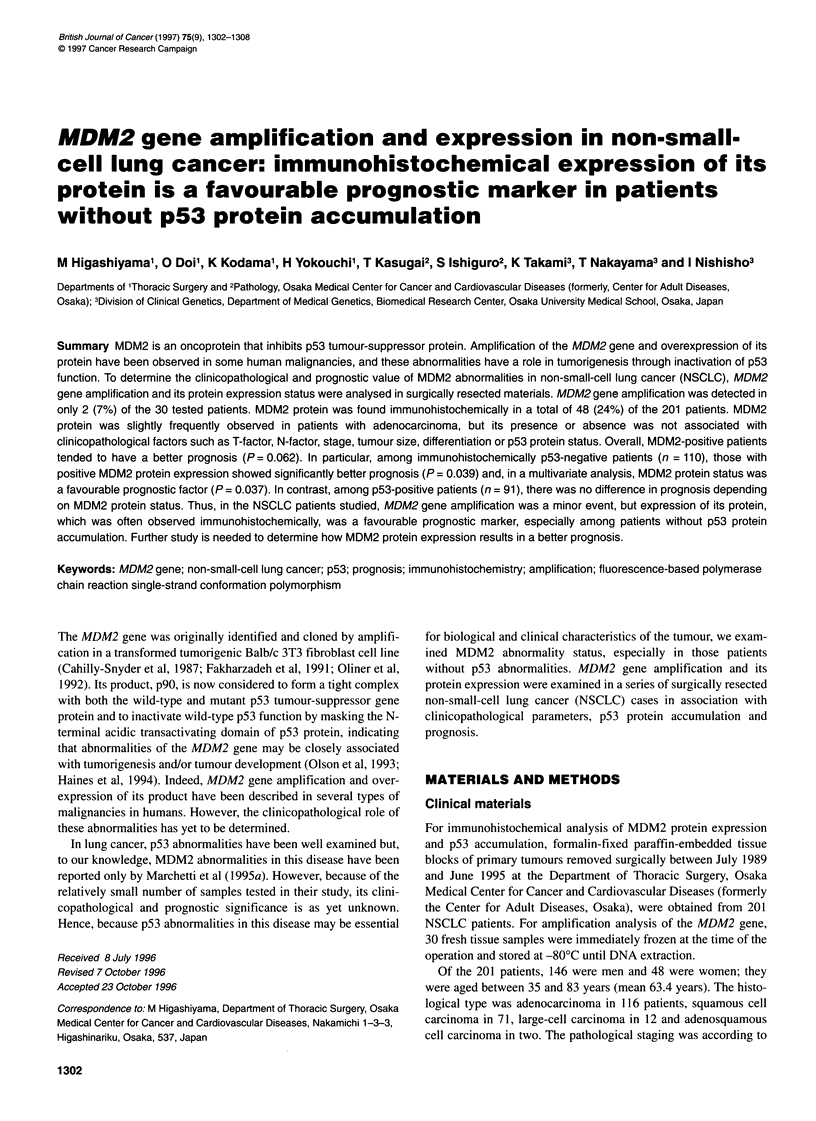

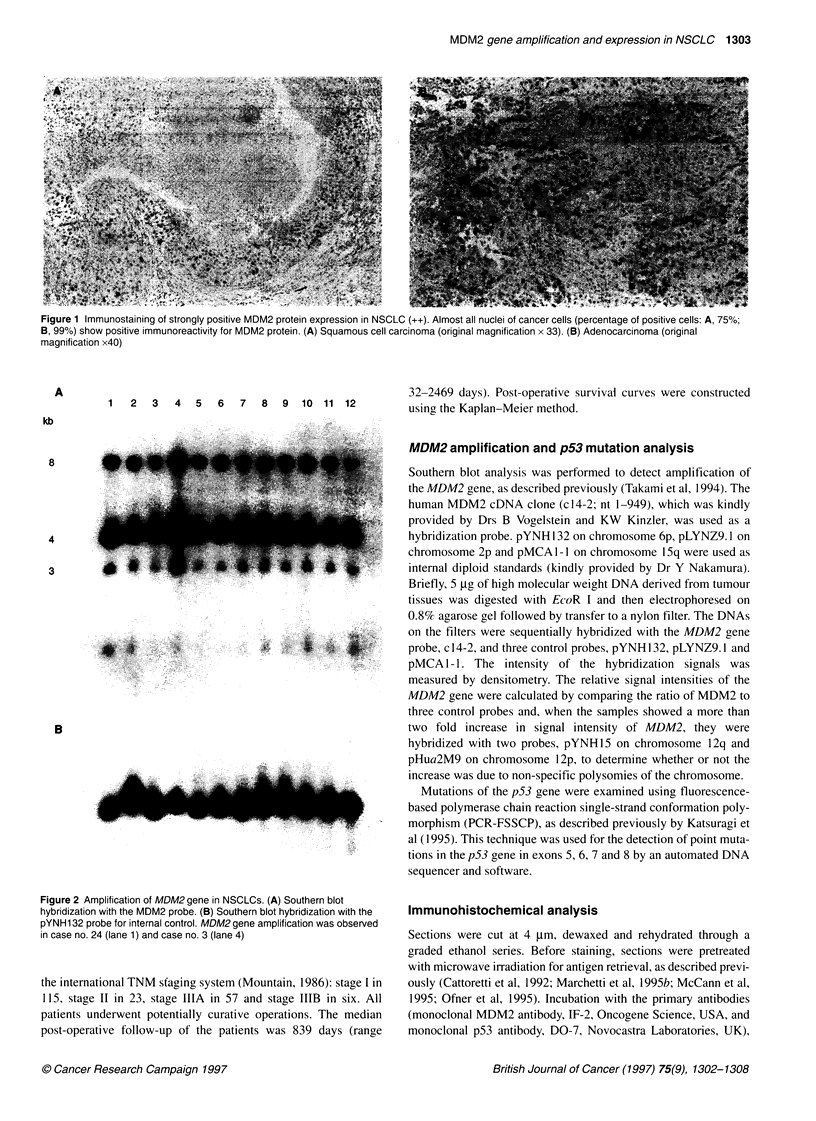

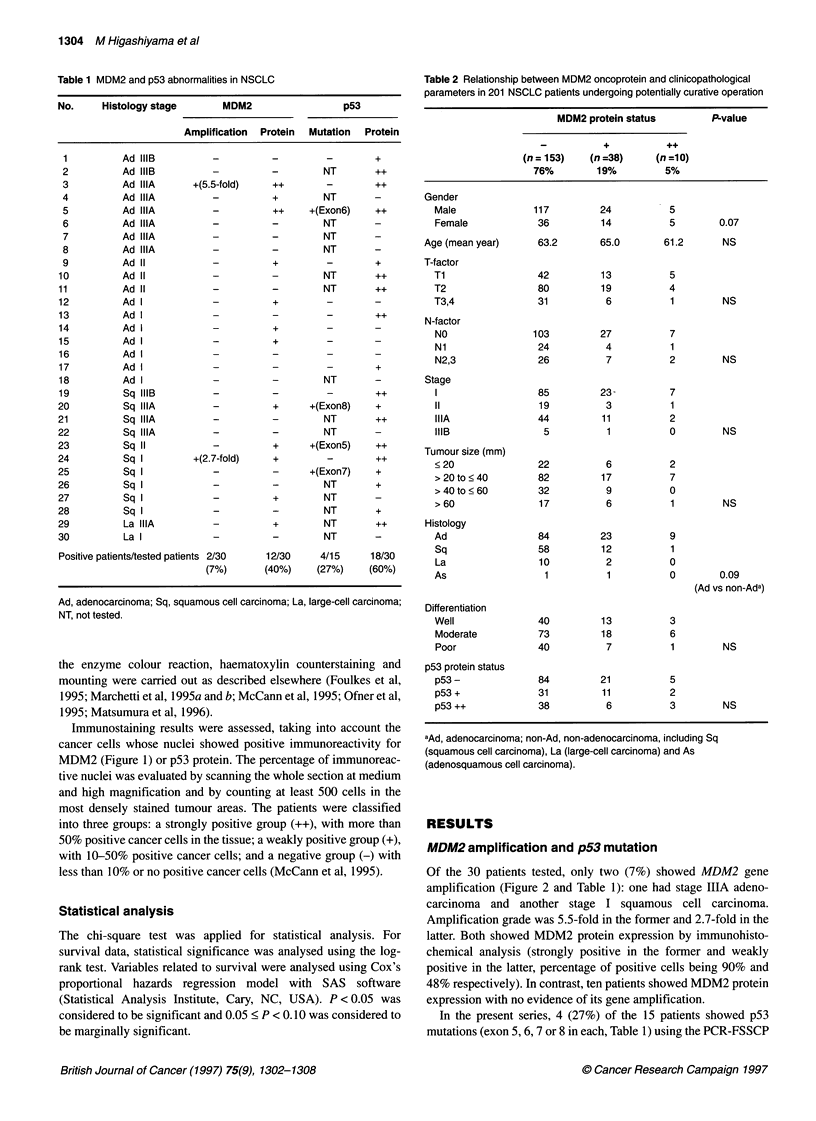

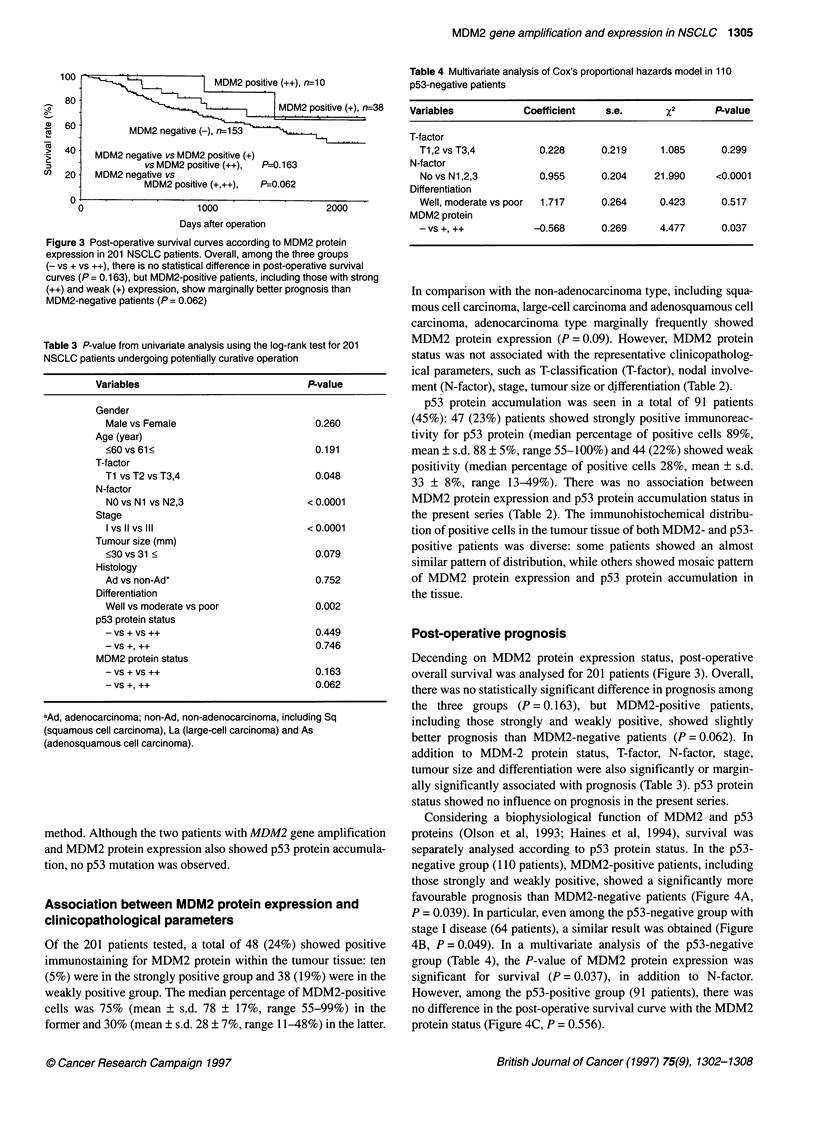

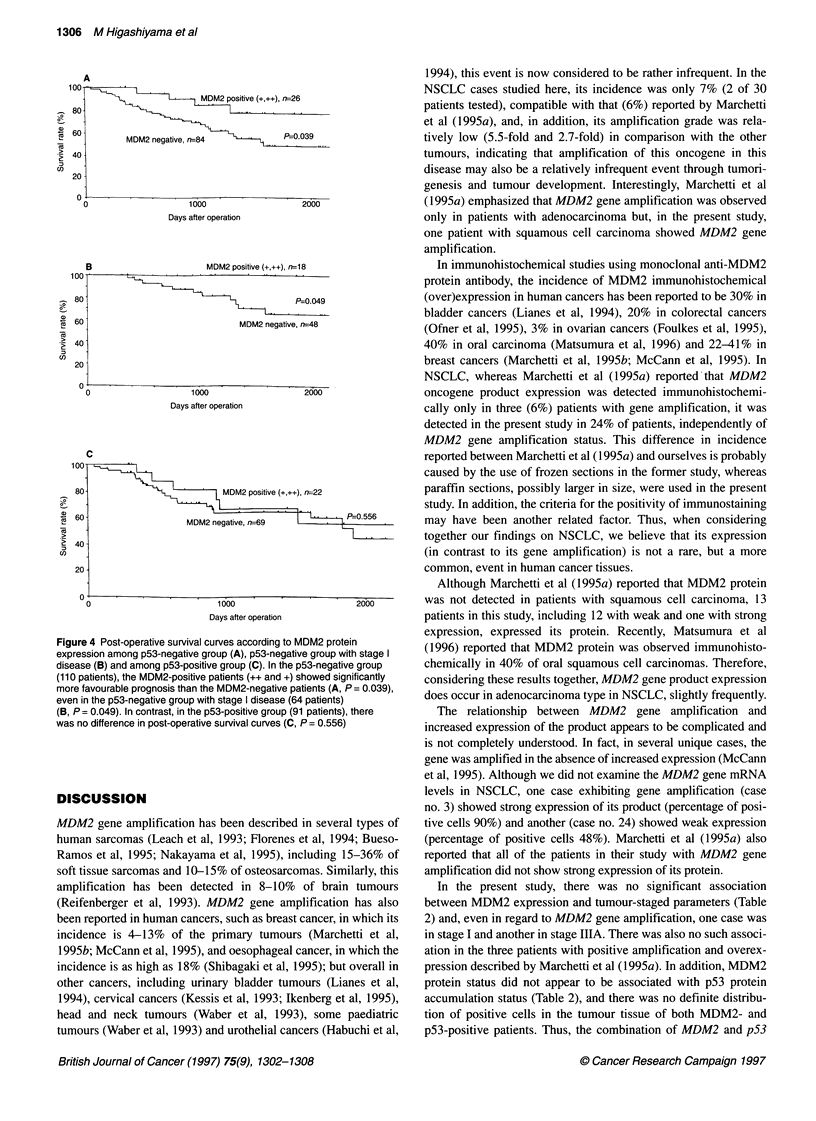

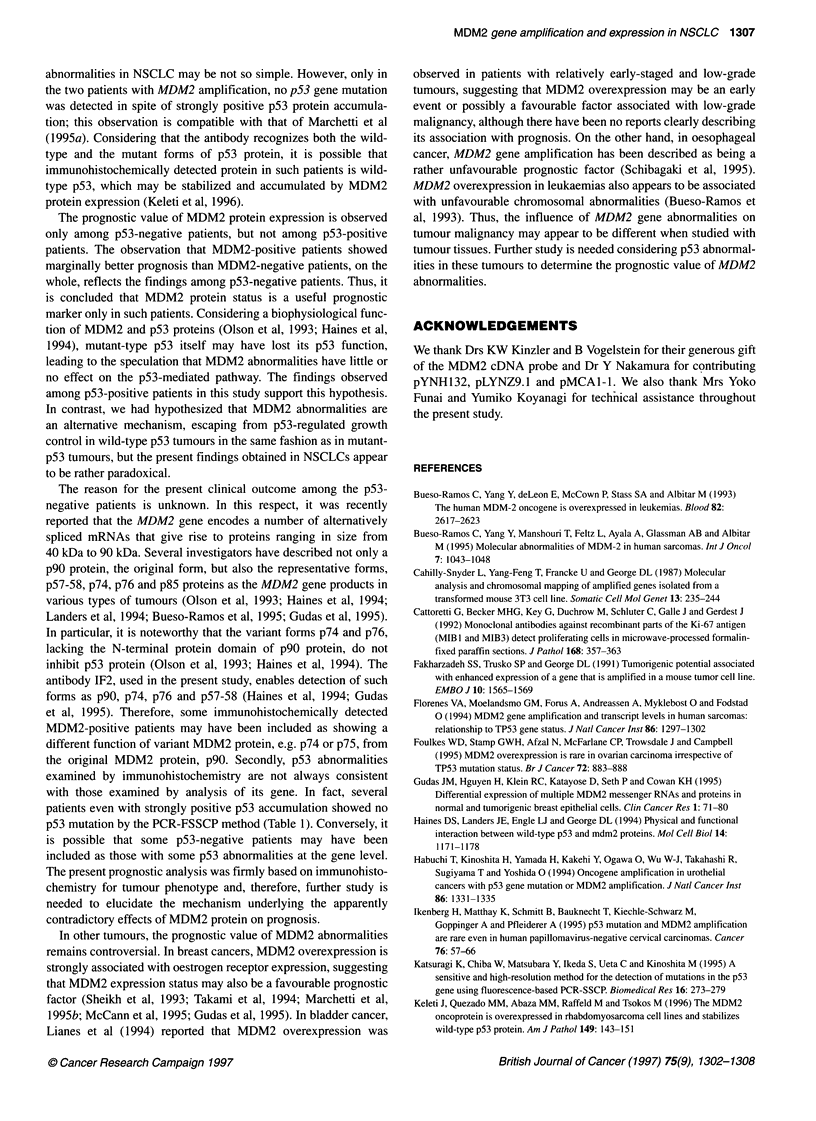

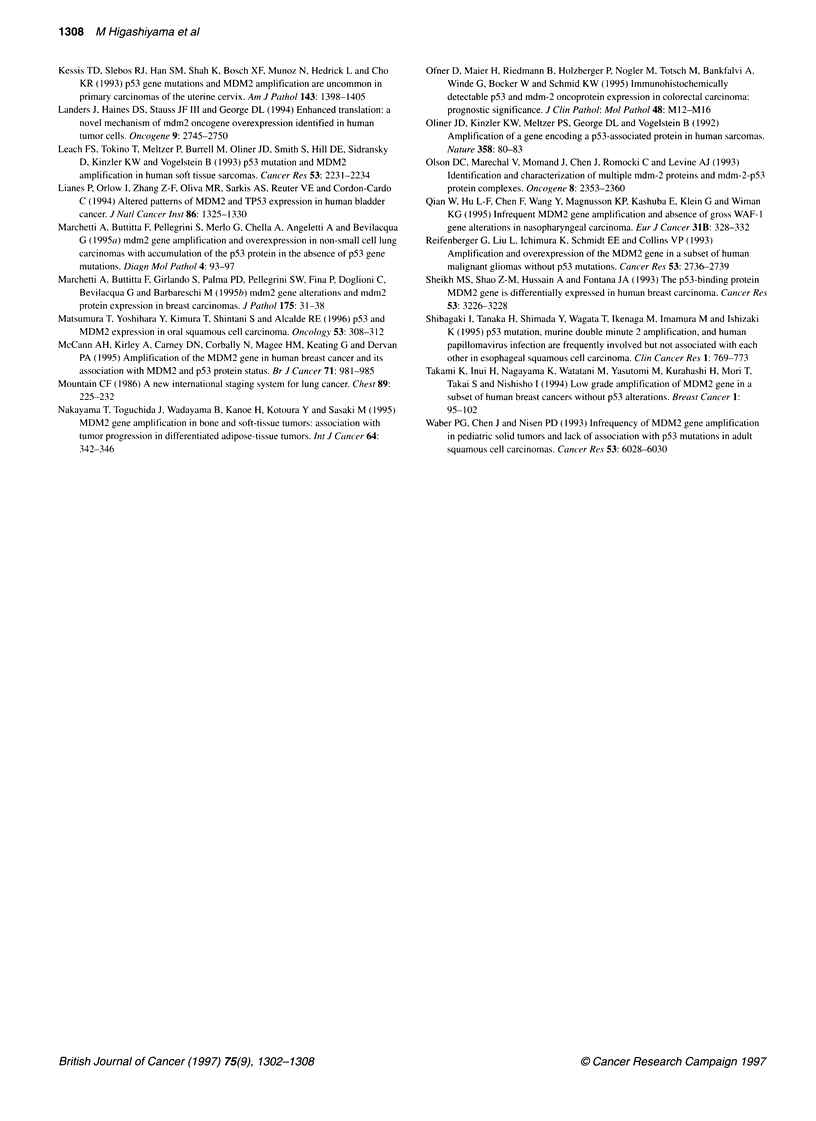


## References

[OCR_00756] Bueso-Ramos C. E., Yang Y., deLeon E., McCown P., Stass S. A., Albitar M. (1993). The human MDM-2 oncogene is overexpressed in leukemias.. Blood.

[OCR_00766] Cahilly-Snyder L., Yang-Feng T., Francke U., George D. L. (1987). Molecular analysis and chromosomal mapping of amplified genes isolated from a transformed mouse 3T3 cell line.. Somat Cell Mol Genet.

[OCR_00771] Cattoretti G., Becker M. H., Key G., Duchrow M., Schlüter C., Galle J., Gerdes J. (1992). Monoclonal antibodies against recombinant parts of the Ki-67 antigen (MIB 1 and MIB 3) detect proliferating cells in microwave-processed formalin-fixed paraffin sections.. J Pathol.

[OCR_00777] Fakharzadeh S. S., Trusko S. P., George D. L. (1991). Tumorigenic potential associated with enhanced expression of a gene that is amplified in a mouse tumor cell line.. EMBO J.

[OCR_00782] Flørenes V. A., Maelandsmo G. M., Forus A., Andreassen A., Myklebost O., Fodstad O. (1994). MDM2 gene amplification and transcript levels in human sarcomas: relationship to TP53 gene status.. J Natl Cancer Inst.

[OCR_00787] Foulkes W. D., Stamp G. W., Afzal S., Lalani N., McFarlane C. P., Trowsdale J., Campbell I. G. (1995). MDM2 overexpression is rare in ovarian carcinoma irrespective of TP53 mutation status.. Br J Cancer.

[OCR_00792] Gudas J. M., Nguyen H., Klein R. C., Katayose D., Seth P., Cowan K. H. (1995). Differential expression of multiple MDM2 messenger RNAs and proteins in normal and tumorigenic breast epithelial cells.. Clin Cancer Res.

[OCR_00802] Habuchi T., Kinoshita H., Yamada H., Kakehi Y., Ogawa O., Wu W. J., Takahashi R., Sugiyama T., Yoshida O. (1994). Oncogene amplification in urothelial cancers with p53 gene mutation or MDM2 amplification.. J Natl Cancer Inst.

[OCR_00797] Haines D. S., Landers J. E., Engle L. J., George D. L. (1994). Physical and functional interaction between wild-type p53 and mdm2 proteins.. Mol Cell Biol.

[OCR_00809] Ikenberg H., Matthay K., Schmitt B., Bauknecht T., Kiechle-Schwarz M., Göppinger A., Pfleiderer A. (1995). p53 mutation and MDM2 amplification are rare even in human papillomavirus-negative cervical carcinomas.. Cancer.

[OCR_00820] Keleti J., Quezado M. M., Abaza M. M., Raffeld M., Tsokos M. (1996). The MDM2 oncoprotein is overexpressed in rhabdomyosarcoma cell lines and stabilizes wild-type p53 protein.. Am J Pathol.

[OCR_00829] Kessis T. D., Slebos R. J., Han S. M., Shah K., Bosch X. F., Muñoz N., Hedrick L., Cho K. R. (1993). p53 gene mutations and MDM2 amplification are uncommon in primary carcinomas of the uterine cervix.. Am J Pathol.

[OCR_00834] Landers J. E., Haines D. S., Strauss J. F., George D. L. (1994). Enhanced translation: a novel mechanism of mdm2 oncogene overexpression identified in human tumor cells.. Oncogene.

[OCR_00839] Leach F. S., Tokino T., Meltzer P., Burrell M., Oliner J. D., Smith S., Hill D. E., Sidransky D., Kinzler K. W., Vogelstein B. (1993). p53 Mutation and MDM2 amplification in human soft tissue sarcomas.. Cancer Res.

[OCR_00845] Lianes P., Orlow I., Zhang Z. F., Oliva M. R., Sarkis A. S., Reuter V. E., Cordon-Cardo C. (1994). Altered patterns of MDM2 and TP53 expression in human bladder cancer.. J Natl Cancer Inst.

[OCR_00856] Marchetti A., Buttitta F., Girlando S., Dalla Palma P., Pellegrini S., Fina P., Doglioni C., Bevilacqua G., Barbareschi M. (1995). mdm2 gene alterations and mdm2 protein expression in breast carcinomas.. J Pathol.

[OCR_00850] Marchetti A., Buttitta F., Pellegrini S., Merlo G., Chella A., Angeletti C. A., Bevilacqua G. (1995). mdm2 gene amplification and overexpression in non-small cell lung carcinomas with accumulation of the p53 protein in the absence of p53 gene mutations.. Diagn Mol Pathol.

[OCR_00861] Matsumura T., Yoshihama Y., Kimura T., Shintani S., Alcalde R. E. (1996). p53 and MDM2 expression in oral squamous cell carcinoma.. Oncology.

[OCR_00865] McCann A. H., Kirley A., Carney D. N., Corbally N., Magee H. M., Keating G., Dervan P. A. (1995). Amplification of the MDM2 gene in human breast cancer and its association with MDM2 and p53 protein status.. Br J Cancer.

[OCR_00874] Nakayama T., Toguchida J., Wadayama B., Kanoe H., Kotoura Y., Sasaki M. S. (1995). MDM2 gene amplification in bone and soft-tissue tumors: association with tumor progression in differentiated adipose-tissue tumors.. Int J Cancer.

[OCR_00880] Ofner D., Maier H., Riedmann B., Holzberger P., Nogler M., Tötsch M., Bankfalvi A., Winde G., Böcker W., Schmid K. W. (1995). Immunohistochemically detectable p53 and mdm-2 oncoprotein expression in colorectal carcinoma: prognostic significance.. Clin Mol Pathol.

[OCR_00887] Oliner J. D., Kinzler K. W., Meltzer P. S., George D. L., Vogelstein B. (1992). Amplification of a gene encoding a p53-associated protein in human sarcomas.. Nature.

[OCR_00897] Qian W., Hu L. F., Chen F., Wang Y., Magnusson K. P., Kashuba E., Klein G., Wiman K. G. (1995). Infrequent MDM2 gene amplification and absence of gross WAF1 gene alterations in nasopharyngeal carcinoma.. Eur J Cancer B Oral Oncol.

[OCR_00901] Reifenberger G., Liu L., Ichimura K., Schmidt E. E., Collins V. P. (1993). Amplification and overexpression of the MDM2 gene in a subset of human malignant gliomas without p53 mutations.. Cancer Res.

[OCR_00906] Sheikh M. S., Shao Z. M., Hussain A., Fontana J. A. (1993). The p53-binding protein MDM2 gene is differentially expressed in human breast carcinoma.. Cancer Res.

[OCR_00911] Shibagaki I., Tanaka H., Shimada Y., Wagata T., Ikenaga M., Imamura M., Ishizaki K. (1995). p53 mutation, murine double minute 2 amplification, and human papillomavirus infection are frequently involved but not associated with each other in esophageal squamous cell carcinoma.. Clin Cancer Res.

[OCR_00919] Takami K, Inui H, Nagayama K, Watatani M, Yasutomi M, Kurahashi H, Mori T, Takai SI, Nishisho I (1994). Low Grade Amplification of MDM2 Gene in a Subset of Human Breast Cancers without p53 Alterations.. Breast Cancer.

[OCR_00923] Waber P. G., Chen J., Nisen P. D. (1993). Infrequency of MDM2 gene amplification in pediatric solid tumors and lack of association with p53 mutations in adult squamous cell carcinomas.. Cancer Res.

